# Biologically meaningful coverage indicators for eliminating malaria transmission

**DOI:** 10.1098/rsbl.2012.0352

**Published:** 2012-05-30

**Authors:** Samson S. Kiware, Nakul Chitnis, Gregor J. Devine, Sarah J. Moore, Silas Majambere, Gerry F. Killeen

**Affiliations:** 1 Biomedical and Environmental Thematic Group, Ifakara Health Institute, PO Box 53, Ifakara, Tanzania; 2 Department of Mathematics, Statistics, and Computer Science, Marquette University, Milwaukee, WI 53201-1881, USA; 3 Department of Epidemiology and Public Health, Swiss Tropical and Public Health Institute, Basel, Switzerland; 4 University of Basel, Basel, Switzerland; 5 Liverpool School of Tropical Medicine, Vector Group, Pembroke Place, Liverpool L3 5QA, UK; 6 Department of Infectious and Tropical Diseases, London School of Hygiene and Tropical Medicine, Keppel Street, London, WCIE 7HT, UK

**Keywords:** GFK insecticides, coverage, malaria, animal, outdoor, mosquito

## Abstract

Mosquitoes, which evade contact with long-lasting insecticidal nets and indoor residual sprays, by feeding outdoors or upon animals, are primary malaria vectors in many tropical countries. They can also dominate residual transmission where high coverage of these front-line vector control measures is achieved. Complementary strategies, which extend insecticide coverage beyond houses and humans, are required to eliminate malaria transmission in most settings. The overwhelming diversity of the world's malaria transmission systems and optimal strategies for controlling them can be simply conceptualized and mapped across two-dimensional scenario space defined by the proportion of blood meals that vectors obtain from humans and the proportion of human exposure to them which occurs indoors.

Indoor residual spraying (IRS) and long-lasting insecticidal nets (LLIN) can dramatically reduce malaria transmission, but will not be sufficient to completely eliminate it from most endemic tropical settings, even if effective drugs and vaccines are available, primarily because of vectors which evade contact with domestic applications of insecticides [1]. At high coverage, most of the protection conferred by these intra-domiciliary measures against malaria transmission by mosquitoes that primarily feed indoors (endophagic) or rest (endophilic) indoors, and primarily feed upon human blood (anthropophagic), occurs at the community level and arises from reduced rates of vector population survival, human blood feeding and reproduction [2]. However, mosquitoes which can rest outdoors (exophilic) or feed outdoors (exophagic), as well as those which feed on animals (zoophagic), are primary malaria vectors in many tropical countries and are obviously less vulnerable to control with insecticides deployed to houses in the form of LLINs and IRS [1,3,4].

Exophagic and zoophagic vectors can therefore comprise an increasingly important fraction of residual transmission in settings where high demographic coverage of LLIN or IRS has successfully suppressed predominant species that primarily feed indoors upon humans [5–11]. For any product conferring personal protection against mosquito bites, it is therefore critical to measure the proportion of human exposure to mosquito bites that otherwise occurs at times when it is practical to use it (*π*) [12]. In the case of LLINs, this definition can be approximately specified as the proportion of normal exposure to mosquito bites upon humans lacking LLINs which occurs indoors when it would be practical to use one (*π_i_*) and measured in the field by weighting the observed indoor (*i*) and outdoor (*o*) biting rates at each period of the night by the surveyed mean proportion of humans that are in these two compartments at that time [13–16]. Where this parameter changes in response to intervention pressure, such changes typically reflect successful control and altered vector population composition [5–11] so the most immediately relevant estimate of this parameter is the baseline value (*π_i_*_,0_) in the pre-intervention scenario (*Ω*) before the effective scale up of those interventions (*Ω* = 0). De facto protective coverage of humans (*C*_h,p_) with LLINs, or any other form of personal protection against indoor exposure, is therefore defined slightly more specifically than before [2,4,12], as the product of crude coverage (*C*_h_; estimated as the reported nightly usage rate) and this proportion of personal human exposure which is practically and directly preventable with an LLIN [2]:1.1
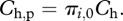


Obviously, the lower the proportion of exposure to a given mosquito population that occurs indoors, the lower will be the impact of LLINs or IRS upon the transmission it mediates, and the more persistent and prominent those populations will be in residual vector systems. Current demographic indicators of coverage for LLINs and IRS often grossly over-represent the degree of insecticidal hazard to which vector mosquitoes are exposed. A conventional demographic view of the current global target of 80 per cent LLIN use among all age groups [17] is presented in figure 1*a*. However, as illustrated in figure 1*b*, only 40 per cent de facto protective coverage of humans is achieved in a scenario with 80 per cent demographic coverage, when only 50 per cent of human exposure occurs indoors.

**Figure 1.**
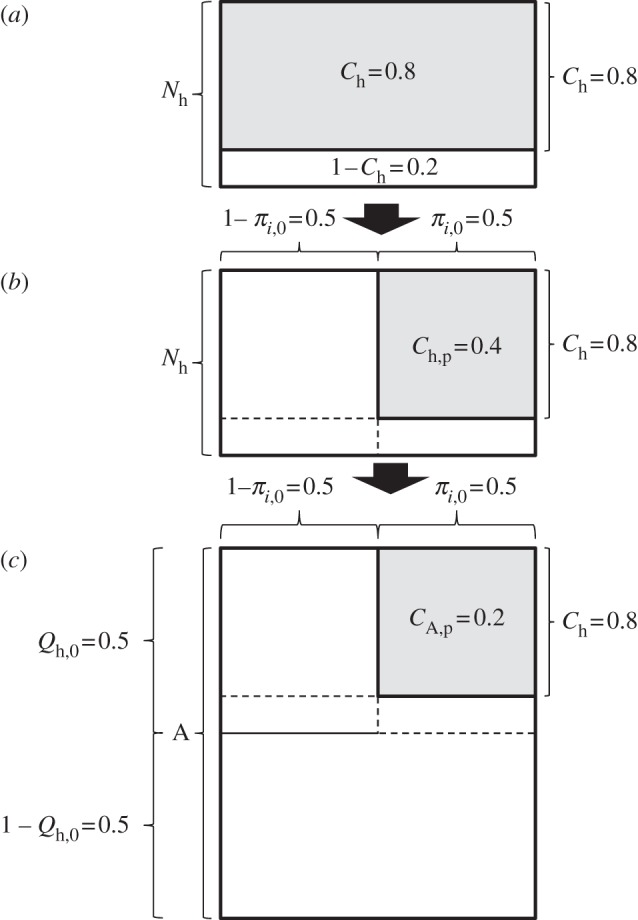
Conceptual schematic of the difference between current demographic indicators of coverage of all humans (*N*_h_) and true biological coverage of all available mosquito blood resources (*A*). In all panels, the proportion considered covered by the stated indicator is represented by the shaded fraction. (*a*) Conventional view of current LLIN/IRS target of 80% crude demographic coverage of all humans while indoor (*C*_h_ = 0.8). (*b*) Protective coverage of humans at all times when either indoors or outdoors (*C*_h,p_; equation (1.1)) where half of human exposure to vectors occurs outdoors (*π_i_*_,0_ = 0.5). (*c*) Biological coverage of all blood resources (*C*_A,p_), equivalent to the covered proportion of all available human and animal blood (*C*_h,p_*A*_h_/*A*; equation (1.2)) in a scenario where half of human exposure to vectors occurs outdoors (*π_i_*_,0_ = 0.5) and animals previously accounted for half of all bloodmeals (*Q*_h,0_ = 0.5).

However, de facto coverage is a biological parameter relating to the coverage of all blood resources that mosquitoes need to thrive and is often even lower than apparent from figure 1*b*. The baseline human blood index (*Q*_h,0_) is defined as the population-wide mean proportion of blood meals that are obtained from humans (*h*), rather than animals, before the introduction of any intervention (*Ω* = 0). This parameter can be readily measured in the field and has long been known as an important determinant of malaria epidemiology and intervention impact [18]. The impact of LLINs or IRS upon the population size and transmission potential of zoophagic vectors is attenuated, even if comprehensive protective coverage of humans is achieved (*C*_h,p_ → 1), because killing them in sufficient numbers to suppress malaria transmission requires high protective coverage of all available blood sources (*C*_A,p_), including animals. This biological indicator of resource coverage is simply the product of the pre-intervention (*Ω* = 0) human blood index (*Q*_h,0_) and the protective coverage of humans (*C*_h,p_) [12]:1.2
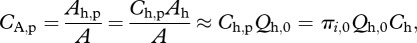
where *A*, *A*_h_ and *A*_h,p_ are the total availabilities or kinetic rates of encounter and feeding and attacking all hosts, all humans and all humans while protected, respectively [2].

Figure 1*c* illustrates how 80 per cent demographic coverage of human users could result in only 20 per cent coverage of the total blood sources available for mosquitoes when the vector obtains half of its blood meals from animals and is equally likely to feed indoors and outdoors. The impact of LLIN or IRS intervention upon vector populations, and therefore the associated selection pressure for heritable resistance traits, are both directly related to this more biologically meaningful coverage indicator with the following simplified form of previous formulations [2]:1.3
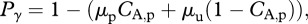
where *P_*γ*_* is the probability of a mosquito surviving all host attack per feeding cycle, while *μ*_p_ and *μ*_u_ represent the mortality probabilities of mosquitoes attacking protected and unprotected hosts, respectively.

The importance of host preference behaviour is best illustrated by the numerous mosquito species that rarely feed on humans, but which do so often enough to sustain stable malaria transmission (0 < *Q*_h,0_ < 0.1) [12], and are primary malaria vectors across much of Asia and the Americas [19]. In stark contrast to settings with strongly anthropophagic vectors [2], LLINs and IRS have far less impact upon malaria transmission by highly zoophagic mosquitoes simply because human blood is of negligible importance to their survival and reproduction [12]. Nevertheless, LLINs and IRS can deliver appreciable community-level protection, for both users and non-users, against transmission by zoophagic vectors where exposure predominantly occurs indoors [12]. This is because humans are the only host for the common malaria parasites (*Plasmodium falciparum* and *Plasmodium vivax*), so the small proportion of a very zoophagic mosquito population that is killed or diverted by these insecticidal products when they encounter humans can be a large proportion of those that actually transmit malaria [12]. As malaria transmission requires at least two feeding contacts between a given mosquito and its human victims, overall minimum immediate impact upon transmission by very zoophagic vectors can be approximated as a very simple squared function of the protective coverage of humans (*C*_h,p_; equation (1.1)) and the entomologically measured estimate of direct personal protective efficacy against biting exposure (*ρ*) [12]:1.4
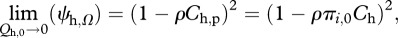
where *ψ*_h,*Ω*_ is the relative rate of exposure to malaria transmission of the average human (*h*) community member immediately after rapidly achieving a specific vector control scenario (*Ω*) defined by the protective coverage and protective efficacy of LLINs or IRS, compared with the average non-user under baseline conditions before scale up [12].

LLINs or IRS are clearly insufficient in themselves to eliminate malaria transmission because de facto protective coverage is attenuated where mosquitoes can readily access blood resources from animals or from humans while they are outdoors (figure 1*c*) [1–4,12,20,21]. As increasing numbers of national programmes attain and sustain high coverage of indoor spaces with IRS or ITNs, complementary strategies are increasingly needed that extend insecticide coverage beyond the house, and indeed beyond humans. Defining, measuring and targeting blood resources other than humans inside houses, which mosquitoes depend upon for survival and which enable them to escape current front-line measures such as LLINs and IRS, are becoming increasingly important. This requires a change in perspective for the responsible communities that have exclusively emphasized human and domestic targets for malaria vector control. Clear understanding of mosquito resource availability, and how to cover them with mosquitocidal measures, is required to eliminate malaria transmission by the diverse array of exophagic, exophilic and zoophagic vectors that exist worldwide. Neglected strategies, such as insecticide-treated clothes, insecticide-treated livestock, repellents, odour-baited traps or larval source management, will be needed to complement LLINs and IRS in order to drive malaria parasite populations to extinction [22]. The development and implementation of these novel technologies will require vastly improved understanding of the ecology of mosquitoes generally, rather than just the handful of highly efficiently anthropophagic vectors that have been the overwhelming focus of research thus far [22].

Fortunately, figure 1*c* represents a simple framework with which the overwhelming diversity of the world's malaria transmission systems, and optimal strategies for controlling them with high coverage (*C*_h_ → 1) of adulticides [2–4,12,20,21], can be readily conceptualized, using only two summary parameters of adult mosquito behaviour that can be readily measured in the field, namely *π_i_*_,0_ [13–16] and *Q*_h,0_ [18,23]. For example, the conclusions of recent modelling analyses for comparing product profiles with a variety of repellent and/or toxic properties in a diversity of vector scenarios, spanning the full range of preferences for feeding upon humans indoor versus outdoor (*π_i_*_,0_) and upon humans versus animals (*Q*_h,0_) [2,4,12], can be mapped across field-measurable two-dimensional parameter space (figure 2), in an intuitive format that is open to experimental evaluation by field epidemiologists, entomologists and ecologists.

**Figure 2.**
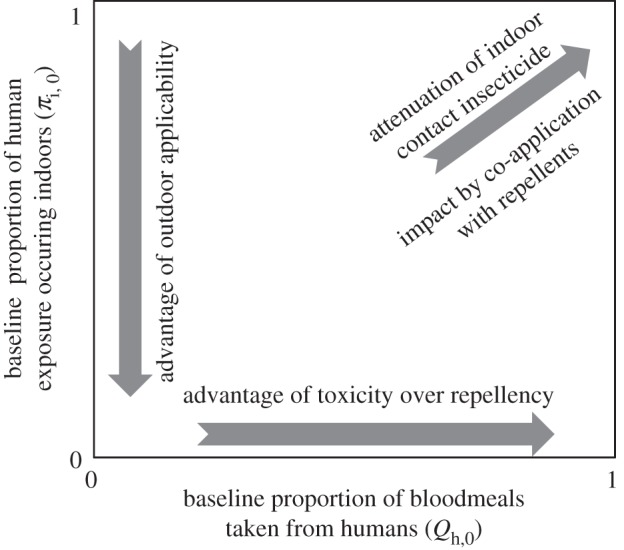
A conceptual summary of the conclusions of recent deterministic modelling analyses [2,4,12,20,21] comparing vector control product profiles with a variety of repellent and/or toxic properties in a diversity of vector scenarios, mapped across the full range of preferences for feeding upon humans indoor versus outdoor (*π_i_*_,0_) and upon humans versus animals (*Q*_h,0_).

## Glossary

*A*: total availability of all hosts: rate at which a single mosquito encounters and attacks all hosts [2,4,12,20,21].
*A*_h_: total availability of all hosts: rate at which a single mosquito encounters and attacks all human hosts.
*A*_h,p_: total availability of all protected hosts: rate at which a single mosquito encounters and attacks all human hosts while protected.
*C*_h_: crude coverage of humans: proportion of people using an LLIN, or similar measure for protection against mosquitoes, each night.
*C*_h,p_: protective coverage of humans: the proportion of all exposure of the human population which is effectively covered by use of protective measures.
*C*_A,p_: protective coverage of all available blood sources: the proportion of all exposure of all available hosts which is effectively covered by use of protective measures.
*μ*_p_ or *μ*_u_: probability that a mosquito which attacks a host will die during the attack upon a protected or unprotected host, respectively.
*N*_h_: number of human hosts.
*P*_γ_: probability that a mosquito survives the host attack event in a single complete feeding cycle.
*π*: proportion of normal exposure to mosquito bites upon humans lacking a given personal protection measure, that occurs at times when it would be practical to use it.
*π*_i,__0_: baseline proportion of normal exposure to mosquito bites upon humans lacking LLINs, which occurs indoors when it would be practical to use one, before any interventions are introduced.
*ρ*: overall proportional personal protection against mosquito bites provide by using a given protective measure.
*Q*_h,0_: baseline human blood index: the proportion of all blood meals which are obtained from humans before any interventions are introduced.
*ψ*_h,*Ω*_: relative exposure of the average human (*h*) to infectious mosquito bites in a given intervention scenario (*Ω*): calculated as a quotient of their exposure divided by that in the absence of any intervention.
*Ω*: intervention scenario defined by coverage level with a specific intervention measure.
